# Reactivity at Bubble Interfaces

**DOI:** 10.3390/mi17070765

**Published:** 2026-06-24

**Authors:** Hongjie An

**Affiliations:** School of Environment and Science, Griffith University, 170 Kessels Road, Nathan, QLD 4111, Australia; hongjie.an@griffith.edu.au

Gas-evolving reactions are ubiquitous in nature and in anthropogenic activities such as energy storage and the manufacturing industries, e.g., chlor-alkali [1] and aluminum production [2,3], electrolysis [4] and photocatalytic water splitting [5,6]. In these reactions, bubbles are produced and attached to the electrode or catalyst surfaces, resulting in overpotentials and reduced reactivity by blocking active surface area and inhibiting mass transfer [4,7,8]. The resulting adverse impact of bubbles is generally seen in photoelectrochemical energy conversion [5,9], carbon dioxide electrolyzers [10], electrodeposition [11] and the charging process of zinc–air batteries [12]. Bubbles can also accelerate catalyst degradation and reduce the efficiency by scattering the incident light in photochemical cells [13,14]. These effects of bubbles have raised concerns and limited their implications as bubbles are naturally ubiquitous in both aqueous and non-aqueous solutions [15].

Although traditional understanding suggests that bubbles mainly act as passive barriers in water splitting and fuel cells, recent studies suggest bubbles can be active players in a complex chemical environment. The interface of surface nanobubbles has been reported to be permeable and they can actively interact with their local surroundings [16]. They remain stable even when they react with the environment [17]. These findings demonstrate that local environment factors, such as surface potential, wettability of the substrate, and saturation level of dissolved gas, determine the fate of bubbles [18]. This is particularly important for applications such as catalysis, water splitting, electrowinning and deposition, and photocatalysis where gas evolves.

To mitigate these adverse effects, recent advances have focused on bubble removal. Technologies, such as magnetic field, surface modification, and acoustic field, have shown positive results. Liang et al. have applied an external magnetic field to mitigate bubble generation during zinc electrodeposition [19]. This Lorentz force-driven approach generated uniform Zn deposition with lower surface roughness and improved performance in alkaline batteries. Similarly, Vensaus et al. reported that the magnetic field significantly enhances mass transfer and reaction efficiency in electrocatalysis, which mitigates concentration polarization and accelerates gas bubble detachment [20]. Ultrasound techniques have been used to effectively accelerate mass transport, reduce bubble residence time, and enhance gas evolution efficiency in water splitting by changing the hydrodynamic behavior of gas bubbles [21]. Kempler et al. have minimized bubble-induced overpotentials by introducing microstructures to the electrode in a water electrolyzer [22].

Beyond these advanced technologies, recent studies suggest that bubbles may actively participate in interfacial reactions. Acceleration of the reaction rates near bubble surfaces has been achieved by the corona effect [23,24]. The corona of the bubbles enhances the localized generation of highly reactive hydroxyl radicals, which further speed up interfacial oxidation kinetics. Vogel et al. [23] employed fluorescence and electrochemiluminescent (ECL) imaging systems to investigate electrochemical performance in the presence of surface bubbles adhering to an indium tin oxide (ITO) electrode. By using a custom three-electrode setup with an ITO working electrode, they found the corona formed at three-phase contact line at a bias of +1.2V vs. standard hydrogen electrode at a basic pH (Figure 1A,B). They concluded that the bubble’s corona accumulates an excess of hydroxide anions and further initiates the oxidation of water-soluble species. They observed a million-fold increase in the lifetime of O_2_•^−^ compared with HO•^−^. In a similar study, Knežević et al. [24] reported that the corona effect of the bubble increases the ECL emission intensity of luminol-based assays five-fold compared with the conventional method using ECL microscopy. They claimed that the new approach overcomes the spatial and temporal limits of conventional ECL by extending the ECL emission range from a few micrometers to 5 mm away from the electrode, while sustaining the luminescence for up to 145 s. These findings suggest micro- and nanobubbles can act as an efficient mass-transport vectors, opening a new window for future catalysis.

However, the interpretation remains controversial. Although these research papers suggest a chemical acceleration via interfacial radical generation due to the corona effect of the bubble, skeptics have raised concerns regarding acceleration of reaction rates due to ECL emission, as bubbles are generally believed to reduce reactivity. Molecular dynamics simulations predicted that surface nanobubbles suppress the reaction rates by restricting water density [26]. Microbubble dynamic study using high-speed imaging on platinum electrodes also showed persistent surface blockage and limited high-current catalytic performance [27]. Layman and Dick [25] observed microbubbles trapped at liquid–liquid phase boundaries far away from the electrode using ECL microscopy, and concluded that the intense light at the edges of a bubble is a result of reflection, scattering due to the curvature, or refraction off the phase boundary rather than localized reaction enhancement at the bubble surface itself (Figure 1C,D).

One possible explanation for the observed corona formation is the electrostatic attraction near the electrodes in the electrochemical environment. Under basic conditions, fluorescent species and hydroxide ions are negatively charged and are therefore instantly driven toward a positively biased anode once the bias is turned on. This results in a local enrichment of reactive species near the three-phase contact line and further increases the oxidation reaction rate. However, the corresponding increase in ECL intensity may not be taken as direct evidence of enhanced electrochemical kinetics, as it may also be influenced by optical effects such as reflection, refraction, and scattering at the curved gas–liquid interface [28]. Notably, different behavior may be expected when the polarity of the electrode is reversed, i.e., when using ITO as a cathode.

Other independent studies have also reported that bubbles can enhance the reaction rates through a mechanism of confined-enhancement mass transport. A research led by Sun at Southeast University has shown that oxygen nanobubbles accelerated the etching reaction rates by over one order of magnitude [29]. They reported that the local etching rate of gold nanorods is significantly enhanced when an oxygen nanobubble is close to a nanorod below the critical distance (~1 nm). They further confirmed that the strong attractive van der Waals interaction facilitates the transport of oxygen through the thin liquid layer to the gold surface. This finding suggests that the acceleration of reaction rates arises from enhanced mass transfer due to tunnelling effect, i.e., confinement-enhanced gas transport. Sun’s findings on etching support the acceleration of catalyst degradation by bubbles. Coincidently, the Western Australia group that discovered the corona effect recently reported a reaction rate enhancement of approximately 10-fold, attributed to the confined thin liquid electrolyte film directly under the bubble footprint due to repulsive van der Waals disjoining forces [30].

In summary, bubbles have traditionally been regarded as adverse factors to gas-evolving reactions due to their impact on mass transport, surface coverage, and energy efficiency. However, recent advances suggest bubbles can also enhance reaction rates under specific interfacial conditions. These controversial observations can be recognized in different mechanisms, such as gas transport, liquid-phase confinement, electrostatic interactions, and optical artifacts. Future research may focus on the fundamentals of reactivity at the bubble interface, as a basic understanding is still lacking. A deeper understanding of this research gap will benefit future design of reaction systems for utilizing or mitigating the impact of bubbles in complex environments, such as electrochemical and catalytic processes.

## Figures and Tables

**Figure 1:**
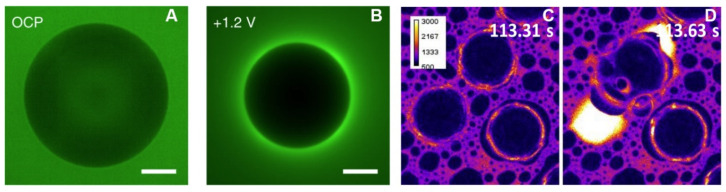
Epifluorescence microscopy images for the detection of HO• around an argon bubble adhering to an ITO electrode. (**A**) For open-circuit potential (OCP). (**B**) Corona appears instantly at bias +1.2 V vs. SHE (reproduced from Ref. [23] with permission from CC BY 4.0). ECL imaging of CO_2_ bubble coalesce on a 1,2-dichloroethane droplet: (**C**) before coalescence and (**D**) after the coalescence of two bubbles (reproduced from Ref. [25] with permission from ACS).

## References

[1] Karlsson R.K.B., Cornell A. (2016). Selectivity between Oxygen and Chlorine Evolution in the Chlor-Alkali and Chlorate Processes. Chem. Rev..

[2] Cooksey M.A., Taylor M.P., Chen J.J.J. (2008). Resistance due to gas bubbles in aluminum reduction cells. JOM.

[3] Toda H., Qu P.C., Ito S., Shimizu K., Uesugi K., Takeuchi A., Suzuki Y., Kobayashi M. (2014). Formation behaviour of blister in cast aluminium alloy. Int. J. Cast Met. Res..

[4] Kempler P.A., Coridan R.H., Luo L. (2024). Gas Evolution in Water Electrolysis. Chem. Rev..

[5] Maeda K., Domen K. (2010). Photocatalytic Water Splitting: Recent Progress and Future Challenges. J. Phys. Chem. Lett..

[6] Zhang B., Wang Y., Feng Y., Zhen C., Liu M., Cao Z., Zhao Q., Guo L. (2024). Coalescence and detachment of double bubbles on electrode surface in photoelectrochemical water splitting. Cell Rep. Phys. Sci..

[7] Angulo A., van der Linde P., Gardeniers H., Modestino M., Fernández Rivas D. (2020). Influence of Bubbles on the Energy Conversion Efficiency of Electrochemical Reactors. Joule.

[8] Moyo O., Sun H., Yang J., Xu X., Shao Z. (2026). Gas bubble formation, effect on electrode performance, and management in water electrolysis. Nano Energy.

[9] Park J., Kim M.J., Kim Y., Lee S., Park S., Yang W. (2025). Insights into Bubble Dynamics in Water Splitting. ACS Energy Lett..

[10] Nwabara U.O., Cofell E.R., Verma S., Negro E., Kenis P.J. (2020). Durable cathodes and electrolyzers for the efficient aqueous electrochemical reduction of CO_2_. ChemSusChem.

[11] Tsai W.L., Hsu P.C., Hwu Y., Chen C.H., Chang L.W., Je J.H., Lin H.M., Groso A., Margaritondo G. (2002). Building on bubbles in metal electrodeposition. Nature.

[12] He Y., Cui Y., Shang W., Zhao Z., Tan P. (2022). Insight into the bubble-induced overpotential towards high-rate charging of Zn-air batteries. Chem. Eng. J..

[13] Bhanawat A., Zhu K., Pilon L. (2022). How do bubbles affect light absorption in photoelectrodes for solar water splitting?. Sustain. Energy Fuels.

[14] Lee J.K., Bazylak A. (2021). Bubbles: The Good, the Bad, and the Ugly. Joule.

[15] An H., Liu G., Atkin R., Craig V.S.J. (2015). Surface Nanobubbles in Nonaqueous Media: Looking for Nanobubbles in DMSO, Formamide, Propylene Carbonate, Ethylammonium Nitrate, and Propylammonium Nitrate. ACS Nano.

[16] German S.R., Wu X., An H., Craig V.S.J., Mega T.L., Zhang X. (2014). Interfacial Nanobubbles Are Leaky: Permeability of the Gas/Water Interface. ACS Nano.

[17] Ouyang L., Ji X., Tan B.H., An H. (2025). Super stable surface nanobubbles under chemical stimuli. Colloids Surf. A.

[18] Ouyang L., Zeng Q., Nguyen N.-T., Tan B.H., An H. (2024). Destabilizing surface bubbles with excessive bulk oversaturation. Colloids Surf. A.

[19] Liang P., Li Q., Chen L., Tang Z., Li Z., Wang Y., Tang Y., Han C., Lan Z., Zhi C. (2022). The magnetohydrodynamic effect enables a dendrite-free Zn anode in alkaline electrolytes. J. Mater. Chem. A.

[20] Vensaus P., Liang Y., Ansermet J.-P., Soler-Illia G.J.A.A., Lingenfelder M. (2024). Enhancement of electrocatalysis through magnetic field effects on mass transport. Nat. Commun..

[21] Cho K.M., Deshmukh P.R., Shin W.G. (2021). Hydrodynamic behavior of bubbles at gas-evolving electrode in ultrasonic field during water electrolysis. Ultrason. Sonochem..

[22] Kempler P.A., Ifkovits Z.P., Yu W., Carim A.I., Lewis N.S. (2021). Optical and electrochemical effects of H_2_ and O_2_ bubbles at upward-facing Si photoelectrodes. Energy Environ. Sci..

[23] Vogel Y.B., Evans C.W., Belotti M., Xu L., Russell I.C., Yu L.-J., Fung A.K.K., Hill N.S., Darwish N., Gonçales V.R. (2020). The corona of a surface bubble promotes electrochemical reactions. Nat. Commun..

[24] Knezevic S., Totoricaguena-Gorriño J., Gajjala R.K.R., Hermenegildo B., Ruiz-Rubio L., Vilas-Vilela J.L., Lanceros-Méndez S., Sojic N., Del Campo F.J. (2024). Enhanced electrochemiluminescence at the gas/liquid interface of bubbles propelled into solution. J. Amer. Chem. Soc..

[25] Layman B.R., Dick J.E. (2023). Through-Space Electrochemiluminescence Reveals Bubble Forces at Remote Phase Boundaries. J. Amer. Chem. Soc..

[26] Wang Z., Yu Y., Qin M., Jiang H., Guo Z., Bai L., Wang L., Zhang X., Zhang X., Liu Y. (2025). Tuning electrode wettability to optimize nanobubble nucleation and reaction rates in water electrolysis. Chem. Eng. J..

[27] Fernández-Vidal J., Vannoy K.J., Bashkatov A., Krug D., van der Heijden O., Lohse D., Koper M.T. (2026). Gas bubble stabilization limits tetraalkylammonium-enhanced hydrogen evolution. ACS Catal..

[28] Arnott W.P., Marston P.L. (1988). Optical glory of small freely rising gas bubbles in water: Observed and computed cross-polarized backscattering patterns. J. Opt. Soc. Am..

[29] Wang W., Xu T., Chen J., Shangguan J., Dong H., Ma H., Zhang Q., Yang J., Bai T., Guo Z. (2022). Solid–liquid–gas reaction accelerated by gas molecule tunnelling-like effect. Nat. Mater..

[30] Vijayakumar V.D., Belotti M., Norret M., Song X., Iannace C., Darwish N., Tabor R.F., Zhang L., Iyer K.S., Ciampi S. (2026). Repulsive Gas–Electrode van der Waals Forces Enable Charge Transfer Reactions under Chemically Modified Bubbles. J. Amer. Chem. Soc..

